# 73936 Developing a Patient-Rated Outcome Measure of Alcohol and Drug Craving: A Systematic Review

**DOI:** 10.1017/cts.2021.505

**Published:** 2021-03-30

**Authors:** Angela M. Haeny, Meghan Morean, Kelly S. DeMartini, Melissa Funaro, Stephanie S. O’Malley

**Affiliations:** 1 Yale School of Medicine; 2 Yale University Medical Library

## Abstract

ABSTRACT IMPACT: The findings from this study will inform the development of an FDA-approved patient-rated outcome measure of drug and alcohol craving that can be used in clinical trials aimed at developing or testing effective treatments for substance use disorder. OBJECTIVES/GOALS: Craving is a potential target of investigative medications to reduce drug use due to the strong link between craving and drug use. We will identify all existing craving measures as the first step for developing an FDA-approved patient-rated outcome measure for use in clinical trials. METHODS/STUDY POPULATION: Following PRISMA guidelines, we will update Rosenberg’s (2009) craving review by conducting a systematic review of all existing published and unpublished measures of craving for alcohol, nicotine, cannabis, opioid, and stimulant use. Electronic database (i.e., Ovid MEDLINE, Embase, PsycINFO, Web of Science, Cochrane), forward, backward, and author searches will be conducted. We will also request unpublished craving measures on major listservs (e.g., Research Society on Alcoholism, the Collaborative Perspectives on Addiction, and the College on Problems of Drug Dependence). All papers included in Rosenberg’s (2009) review through September 2020 will be included. RESULTS/ANTICIPATED RESULTS: The findings from this review will provide a comprehensive summary of the construct of craving and its hypothesized and tested domains. This review will elucidate whether the literature suggests there are components of craving unique to alcohol, nicotine, cannabis, opioid, and/or stimulant use, and whether there are key elements of craving common across the disorders. Therefore, these findings will inform whether a single patient-rated outcome measure of craving can be developed for use across substances or if unique patient-rated outcome measures of craving need to be developed for each substance. DISCUSSION/SIGNIFICANCE OF FINDINGS: While many different measures of craving exist, none have gone through the developmental steps required to qualify as an FDA-approved patient-rated outcome measure on which drug treatment labeling can be based. Completing this systematic review is the first step in this process.

